# Microglia-Synapse Pathways: Promising Therapeutic Strategy for Alzheimer's Disease

**DOI:** 10.1155/2017/2986460

**Published:** 2017-04-03

**Authors:** Jingdun Xie, Haitao Wang, Ting Lin, Bingtian Bi

**Affiliations:** ^1^Department of Anesthesiology, Sun Yat-sen University Cancer Center, State Key Laboratory of Oncology in South China, Collaborative Innovation Center of Cancer Medicine, Guangzhou 510060, China; ^2^Guangdong Provincial Key Laboratory of New Drug Screening, School of Pharmaceutical Sciences, Southern Medical University, Guangzhou 510515, China; ^3^Department of Nephrology, Guangdong General Hospital, Guangdong Academy of Medical Sciences, Guangzhou 510080, China; ^4^Department of Clinical Trial Center, Sun Yat-sen University Cancer Center, State Key Laboratory of Oncology in South China, Collaborative Innovation Center of Cancer Medicine, Guangzhou 510060, China

## Abstract

The main hallmarks of Alzheimer's disease (AD) are extracellular deposits of amyloid plaques and intracellular accumulation of hyperphosphorylated neurofibrillary tangles (tau). However, the mechanisms underlying these neuropathological changes remain largely unclear. To date, plenty of studies have shown that microglia-mediated neuroinflammation contributes to the pathogenesis of AD, and the microglia-synapse pathways have been repeatedly identified as the crucial factor in the disease process. In this review, evidences from microglia and synapse studies are presented, and the role of microglia in the pathogenesis of AD, the contributing factors to synapse dysfunction, and the role and mechanisms of microglia-synapse pathways will be discussed.

## 1. Introduction

Alzheimer's disease (AD) is a progressive neurodegenerative disorder in the elderly that is characterized by progressive cognitive dysfunction [1], synaptic degeneration, neuronal loss, accumulation of amyloid-beta (A*β*) and neurofibrillary tangles (tau), and increased neuroinflammation which are the hallmarks of AD in the brain [2, 3]. During the past 30 years, drug development for AD aimed at various potential targets has experienced tremendous, global setbacks, which have been persistent enough to make the efforts to find anti-AD drugs appear to be an ineffective strategy; for example, the failure rate of anti-A*β* drugs in clinical trials is approximately 100% [4–8]. Discovering new disease mechanisms and investigating the disease network of AD will aid in the identification of the pathogenesis of AD and potential treatments for this disease. Recently, many studies have shown that microglia-mediated neuroinflammation contributes to the pathogenesis of neurodegeneration and AD [9–12], and this neuroinflammation has been identified as a risk factor for AD [13–16], but the underlying mechanisms of neuroinflammation in AD remain unclear. It has been repeatedly shown that microglial activation is associated with synaptic dysfunction, and this response can have harmful consequences for neuronal function that may eventually lead to cognitive and behavioral deficits. Synaptic plasticity is one of the most important factors that regulates the formation of A*β* [17] and the long-term potentiation (LTP) pathways in AD. However, most studies have focused on the relationship between microglia and A*β* and tau, and little is known about microglial activities in early stage AD; microglia-synapse interactions especially need to be clarified. In this review, evidences from microglial studies are presented, and the role of microglia in the AD brain, as well as microglia-synapse pathways, will be discussed.

## 2. Microglia in Healthy and AD Brains

Microglia are the predominant immune cells in the brain, constantly surveying their microenvironment to detect potential threats and regulating the response to neuroinflammation by rapidly secreting signaling molecules, such as neurotransmitters, cytokines, and extracellular matrix proteins [18, 19]. In contrast to the healthy, young brain, the aging brain contains activated microglia accompanied by elevated levels of chemokines and cytokines, such as interleukin-1 beta (IL-1*β*), interleukin-6 (IL-6), and Tumor Necrosis Factor-Alpha (TNF-*α*) [20]. Interestingly, microglia in the brains of aged mice exhibit a distinctly different expression profile and response to lipopolysaccharide (LPS) compared to those of young mice [21]. Upregulation of the major histocompatibility complex II (MHCII) and complement receptor 3 (CR3) in aging brains indicates the aggregation of microglia [22, 23]. The mechanism of these age-dependent changes in microglial function remains elusive. Since similar changes are observed in neurodegenerative disease, the mechanism needs to be studied intensively.

In addition to modulating the inflammatory responses in the brain, microglia also influence neuronal function [24, 25]. Studies have shown that microglia are highly dynamic and make efficient contact with neurons; they can also rapidly cruise throughout the whole brain parenchyma [26]. Microglia have receptors for both neurotransmitters and neuronal modulators, indicating that microglia can respond to neurons [27]. Microglia exert effects upon synapses and neuronal circuits by promoting synapse formation [28], as evidenced by the following findings: genetic depletion of brain-derived neurotrophic factor from microglia largely reduced synaptic spine formation [29] and genetic ablation of microglia decreases spine density, excitatory synapses, and relative connectivity in layer 4 neurons [30], which provides new insights into microglia-mediated neuronal circuit development, with potential implications for the development of brain dysfunction.

Previous research indicates that activated microglia can be categorized into a proinflammatory M1 phenotype and an immunosuppressive M2 phenotype. M2 microglia with A*β* phagocytic capability in APP/PS1 transgenic mice at 6 months of age can switch to M1 phenotypes at 18 months of age, in accordance with the levels of soluble A*β* oligomers [31]. Microglia in AD patients may exhibit mixed activation phenotypes. Colton et al. probed cortical tissue from patients with AD for markers of alternative activation and found increased Arginase-1 (Arg1), Cluster of Differentiation 206 (CD206), Chitinase-3-like protein 1 (Chi3 l1), Chitinase-3-like protein 2 (Chi3 l2), and TNF-*α*, with unchanged expression of inducible nitric oxide synthase (iNOS) and IL-1*β* [32]. An early decrease in mitochondrial uncoupling protein-2 (UCP2) was induced by the M1 stimulus LPS and also responds to M2 stimuli, as indicated by its persistent upregulation by IL-4. In UCP2-silenced microglia, IL-4 failed to induce the M2 genes mannose receptor 1 and IL-10 and reduce M1 genes TNF-*α* and iNOS, indicating that UCP2 is crucial in the microglial activation process with opposing regulation of M1 and M2 responses and the redirection of microglial response toward the protective phenotype in AD [33].

The inflammasome signaling pathway has been described in AD; A*β* can activate the nucleotide-binding and oligomerization domain-like receptor family pyrin domain containing 3 (NLRP3) inflammasome in microglia [34], which is necessary for the secretion of proinflammatory cytokines. Recently, Heneka et al. demonstrated that the activation of the NLRP3 inflammasome plays a critical role in AD pathogenesis by mediating the chronic inflammatory response, while inhibition of NLRP3 almost completely protected against memory impairment and decreased A*β* deposition in APP/PS1/NLRP3^−/−^ transgenic mice [35]. Interestingly, the activation of the cerebral, endogenous NLRP3 inflammasome was restricted to the microglia surrounding the plaques, and the microglial-specific disruption of the NLRP3 inflammasome skewed the microglial phenotypes toward M2, which could potentially reduce A*β* load and protect against cognitive decline [35], suggesting that microglia-specific activation of the NLRP3 inflammasome is important for AD pathogenesis. In addition, using microglia-specific atg7-deficient mice, researchers demonstrated that the A*β*-induced NLRP3 inflammasome is regulated by microglial autophagy associated with A*β* clearance, which suggests that a therapeutic strategy that enhances microglial autophagy could interfere with NLRP3-induced inflammation in AD [36].

Toll-like receptors (TLRs) are important molecules for initiating immune responses, and the expression of TLR4 has been verified on microglia [37]. TLR4 can mediate microglial activation, such as phagocyte activity, proinflammatory cytokine release, and ROS production, in response to C-terminal-truncated synuclein [38]. Current studies have demonstrated that microglial activation is dependent on TLR4 expression in AD [39]. TLR4 has specifically attracted attention in several inflammatory diseases, including cerebral diseases [40]. Both pharmacological inhibition of TLR4 and TLR4 knock-out in mice induce neuroprotection in experimental stroke [41, 42]. In neuroinflammatory conditions, there is release of endogenous galectin-3 (Gal3), which subsequently binds to and stimulates microglial TLR4 and induces the M1 phenotype in microglia in the brain [43]. Moreover, both activated microglia and A*β* oligomers are implicated in neuronal and cognitive dysfunction in AD. A recent study revealed that the anti-inflammatory drug indomethacin and an IL-1*β*-receptor antagonist prevented A*β* oligomer-mediated memory impairment. Administration of the TLR4-receptor antagonist or knock-out of the TLR4 gene in mice abolished the effects of A*β* oligomers on cognitive function, providing novel evidence of the crucial role of TLR4 in microglial-associated neuroinflammation and AD pathogenesis [44].

Recently, microRNAs (miRNAs) have emerged as novel, gene-regulatory elements during aging. miRNAs consist of 18–22 nucleotides and are noncoding RNAs that work posttranscriptionally to shape the cellular transcriptome. To date, more than 2500 human miRNAs have been identified, but only highly selective miRNAs appear to be enriched in the CNS. Understanding the functions of miRNAs in the brain will provide further insight into the process of neurodegenerative diseases, including AD. miR-124 is a key regulator of microglia quiescence in brain, and miR-124-knockdown microglia can activate themselves by inhibiting the transcription factor CCAAT/enhancer-binding protein-a (C/EBP-a) and its downstream target PU.1 [45]. Interestingly, a recent study demonstrated that miRNA-146a targets several inflammatory messenger RNAs, including those encoding complement factor-H, a crucial inhibitor of the cerebral inflammatory response [46]. Indeed, upregulation of miR-146a and concomitant downregulation of complement factor-H were observed in the brain of transgenic AD mice [47]. A potential role for miR-181c has been found in the regulation of one of its targets, TNF-*α*, in which the repressive function of miR-181c is attenuated by microglia-mediated neuronal apoptosis by suppressing TNF-*α* [48]. miR-27a was also observed to negatively modulate LPS-induced production of inflammatory cytokines such as IL-1*β*, IL-6, TNF-*α*, and nitric oxide in microglia, independent of TLR4 and interleukin-1 receptor-associated kinase 4 (IRAK4), suggesting that miR-27a is associated with microglial activation and the inflammatory response [49].

## 3. Microglia-Mediated Synapse Loss in AD

Most patients with AD have the sporadic form of the disease, which arises from defined genetic and external environmental factors. These factors do not directly affect A*β* formation but destroy the clearance mechanisms instead [50]. Similar to macrophages in CNS, microglia are mainly responsible for phagocytosis and clearance of cellular fragments or misfolded proteins, including A*β* and tau [51, 52], and research shows that microglia are dystrophic rather than activated in the AD-affected CNS [53]. While inflammatory processes regulated by the cerebral immune system are considered to be features of AD pathology, the role of the microglial response in AD has become an attractive area of research. To address the above knowledge gaps, researchers generated APP/PS1 mice deficient for interleukin-10, which is mainly secreted by microglia in central nervous system (CNS), and showed a striking exacerbation of amyloid pathology and cognitive impairment due to impaired microglial motility and phagocyte capacity [54].

Synapse loss is an early event in the course of AD, and the correlation between synapse density and the degree of cognitive impairment, as measured by the Mini Mental State Examination test and verbal fluency test, is well established in patients with AD [55, 56]. Interestingly, spatial learning and memory impairment, an early clinical feature of AD, is caused by synaptic dysfunction rather than neuronal loss in AD transgenic mice [57]. Functional and structural changes of synapses are thought to occur early in the pathogenesis of AD. Studies reported that synapse loss occurs in the dentate gyrus (DG) of the brains of AD patients [58, 59]. Other studies found that the synaptic densities in the DG of Tg2576 transgenic mice have increased [60] or decreased in APP/PS1 mice [61]. In a transgenic mouse model of AD, no changes in synaptophysin expression occur, but the outer molecular layer (OML) of the DG exhibits a significant decrease in synaptic density [61]. In addition, A*β* oligomers cause large dendritic spines to reduce their cytoplasm volume and simultaneously remodel their postsynaptic elements in the neuropil of APP/PS1 mice [62].

In an AD mouse model with A*β* deposits, researchers have noted that the soluble forms of amyloid oligomers have an important role in initiating the disease, possibly by targeting synapses [63]. Using two-photon microscopy (TPM) in vivo imaging, extensive synaptic abnormalities associated with dense core amyloid plaques were observed in mice crossbred from transgenic AD mice and mice expressing green fluorescent protein (GFP) in subsets of neurons [64]. Furthermore, in an AD mouse model of tauopathy, prominent microglial activation coincided with synapse loss in hippocampus and impaired synaptic function prior to the onset of fibrillary tau tangles [65, 66].

Mitochondria, the main source of reactive oxygen species (ROS), play a crucial role in the events leading to ROS release, and mitochondrial dysfunction can be one of the key initiating factors of AD [67]. According to the “mitochondrial cascade” hypothesis [68], decreased ATP synthesis and ROS detoxication result in the overproduction of A*β* and promote tau protein hyperphosphorylation, synapse dysfunction, and apoptosis, effectively exacerbating the neurodegenerative processes. The ensuing consequences of mitochondrial dysfunction are impairment of the cell membrane by free radicals and neuroinflammation, leading to synaptic transmission dysfunction, increased glutamate release from presynaptic terminals, and decreased plasticity in synaptic contacts [20, 69].

Imaging studies have shown that microglia constantly contact synapses and modify synaptic connections in the healthy brain [29, 70–72]. Abnormal microglia, especially with altered expression of immune-related receptors, cause synaptic and wiring dysfunction in the brain during development [71, 72], supporting the role of microglia in pruning synaptic connections. A recent study using the APP/PS1 transgenic mouse model discovered the existence of a new myeloid-cell phenotype, called “dark microglia,” which are distinct from the “normal” microglia described at the ultrastructural level. Dark microglia are rarely present in the healthy brain but are abundant in the brains of APP/PS1 transgenic mice. They strongly engage in the engulfment of dendritic spines, axon, and synapses, which indicates that they participate in the remodeling of neuronal circuits in AD [73]. Dark microglia stain for IBA1 and GFP in the brain tissue of fractalkine receptor 1- (CX3CR1-) GFP mice and strongly express CR3 and microglia-specific 4D4 in the processes that engulf synaptic elements [74]. Identification of key markers of dark microglia is a topic worthy of further investigation, as these cells may mediate synapse degeneration in the pathogenesis of AD.

## 4. Possible Mechanisms of the Microglia-Synapse Pathways in AD

Microglial interactions with synapses in AD obviously affect the maturation of synapses and neuronal viability; elucidating the possible mechanisms of microglia-synapse pathways may provide further insights into immune system regulation of neuronal circuit development in the AD brain and novel approaches for AD treatment.

Identifying genetic interactions in data obtained from genomewide association studies (GWAS) can help develop an understanding of the genetic changes in diseases. In recent years, GWAS have revealed a series of new susceptibility genes for AD, including ABCA7, BIN1, CR1, CD2AP, CD33, CASS4, CELF1, CLU, EPHA1, FERMT2, HLADRB5/HLA-DRB1, INPP5D, MS4A6A, MEF2C, NME8, PICALM, PTK2B, SLC24A4/RIN3, SORL1, TREM2, and ZCWPW1 [9, 10, 13, 75–82]. Among these genes, BIN1, CD33, and TREM2 are microglia-specific genes [83, 84], suggesting that microglia are strongly associated with AD pathogenesis.

BIN1, also known as bridging integrator-1, appears in the microglial signature in GWAS, indicating that it may affect the immune system in AD. Human Bin1/amphiphysin 2 and amphiphysin 1 are members of the amphiphysin family, and amphiphysin 1 and amphiphysin 2 share 50% amino-acid sequence homology. As the second-most prevalent susceptibility gene for late-onset AD, BIN1 has similar domain structure to amphiphysin 1 [85], which is strongly implicated in synaptic vesicle endocytosis [86].

CD33 is a transmembrane protein that regulates innate immunity in CNS. Increased expression of CD33 in microglia has been observed in the AD brain, and the numbers of CD33-positive microglia were positively correlated with insoluble A*β*42 levels, which is reversed in APPSwe/PS1DE9/CD33−/− mice [87], suggesting the potential of CD33 inhibition in microglia as a treatment for AD, but the relationship between CD33 and microglia-synapse interactions remains elusive.

TREM2, also referred to as triggering receptor expressed on myeloid cells 2, is uniquely expressed by microglia in the brain [88] and has been identified as a novel risk target for AD. Although the natural ligands of TREM2 are unknown, TREM2 activation was verified to regulate the activity of microglia, including cytokine secretion and autophagy [89], and to maintain microglial survival [90]. TREM2 overexpression reduced cognitive impairments and ameliorated neuronal and synaptic loss as well as tau hyperphosphorylation in P301S tau transgenic mice. Meanwhile, TREM2 reduced the expression of proinflammatory cytokines as well as the activity of tau kinase [91]. Interestingly, TREM2 overexpression in the brains of wild-type mice did not appear to improve spatial learning and memory, suggesting that the inhibition of neuronal and synaptic loss by TREM2 overexpression was likely caused by the ascending tau hyperphosphorylation pathway. In future studies, the effects of TREM2 on the synaptic pruning pathways in AD should be elucidated.

Elimination of abnormal synapses is critical for neuronal circuit development. A previous study [70] showed that microglia contribute to synaptic pruning during development, and PSD95 (postsynaptic component) or SNAP25 (presynaptic component) was coexpressed in microglia in the hippocampus, indicating that microglia engulf synaptic components. Fractalkine (CX3CL1)/CX3CR1 and the classical complement system are considered mediators of microglia interactions with synapses [70]. CX3CR1, specifically expressed on the surface of microglia, appears to play a crucial role in synaptic pruning. Fuhrmann et al. [92] crossed AD mice with transgenic mice (3XTg, CX3CR1-GFP, and Thy1-YFP) lacking the CX3CR1 receptor (3XTg:CX3CR1−/−:Thy1-YFP) and showed significant synapse loss in addition to attenuated microglial migratory velocity, indicating a critical role for the CX3CR1 chemokine receptor in microglia-mediated neuronal circuits in AD.

The classical complement proteins C1q and C3 localize to the synapse and mediate synapse elimination by microglia [93, 94]. C1q, the initiating protein of the classical complement cascade, was increased and associated with synapses before obvious plaque deposition in hAPP transgenic mice. Inhibition of C1q, C3, or the microglial CR3 reduces the number of microglia, as well as the extent of synapse loss in those mice at 3 to 4 months of age. Moreover, microglia in adult brains engulf synaptic material in a CR3-dependent process when exposed to soluble A*β* oligomers [25]. These findings suggest that the complement-dependent pathway and microglia (which prune synapses during development) mediate synapse loss in AD.

A very early loss of synaptic plasticity in vivo was reported in APP/PS1 transgenic mice; these mice exhibit defective ocular dominance plasticity (ODP) in visual cortex during the critical period in development [95]. This observation directly contrasts with results from mice lacking paired immunoglobulin-like receptor B (PirB) associated with synapses, in which ODP is enhanced during the development of the visual cortex [96]. A recent study showed that human PirB and its ortholog leukocyte immunoglobulin-like receptor B2 (LilrB2) are receptors for A*β* oligomers. Cofilin is recruited and activated by PirB in an A*β*-dependent manner in vivo and in vitro and is altered in the human AD frontal cortex. In mice, the deleterious effect of A*β* oligomers on long-term potentiation in hippocampus required PirB, and, in the APP/PS1 transgenic model of AD, PirB not only contributed to the memory deficits present in adult mice but also mediated loss of synaptic plasticity in the juvenile visual cortex [97]. PirB is also expressed in neurons [96] and modulates neurite outgrowth and neuronal plasticity by interacting with three axonal outgrowth inhibitors, Nogo, OMgp, and MAG. It was also shown that genetic deletion of PirB can dampen the inhibitory effects of myelin-associated inhibitory proteins (MAIs) [98]. Additionally, PirB mutant mice showed greater visual cortical plasticity following injury compared with control mice [99]. These findings suggest that PirB may play an inhibitory role in neurite growth following CNS injury. Therefore, it may be possible to enhance axonal regeneration, synaptic plasticity, and subsequent motor recovery by antagonizing PirB.

Mitochondrial dysfunction is one of the early features in the AD brain [100, 101]. Studies have highlighted the role of mitochondrial A*β* accumulation in the pathogenesis of AD. Accumulation of A*β* in the mitochondria precedes the extracellular A*β* deposition in the AD brain and increases with age and is also associated with early onset of loss of synapses, synaptic damage, and mitochondrial oxidative damage [102–104]. CcO is a key enzyme involved in complex IV of the mitochondrial respiratory electron transport chain. CcO reduction is well documented at various stages of AD, including the early mild cognitive-impairment stage [105, 106]. A recent study found a significant reduction in CcO activity in transgenic mAPP mice at the age of 8-9 months compared to wild-type mice. Similarly, ATP levels were significantly reduced in transgenic mAPP mice, indicating dysfunctional mitochondria and energy metabolism, and mitochondrial dysfunction also exacerbates learning and memory deficits, as demonstrated by mAPP mice with impairment of spatial learning and memory [107].

## 5. Microglia-Synapse Interactions in the Early Stages of AD

Synapse degeneration is an early, important event in AD; however, whether microglia participate in these early stages of AD pathogenesis and interact with synaptic dysfunction remains unclear. Hong et al. have demonstrated that resident microglia in the adult CNS phagocytose synapses when challenged by oligomeric A*β*. Interestingly, depending on microglial CR3 expression, microglia act as early mediators of synapse degeneration or loss that occur in AD mice at 3 to 4 months of age, prior to plaque deposition, providing new evidence for how synapses are lost in early stage of AD. Microglia could be a potential early therapeutic target in AD involving synaptic loss and cognitive impairment.

Increased microglial activation and neuroinflammation appear to accompany both A*β* and tau pathology [108, 109]. Since TREM2 is prominently involved in the activation of microglia, soluble TREM2 (sTREM2), which can be detected in cerebrospinal fluid (CSF), is a potential candidate marker for tracking the progression of AD. Some studies [110, 111] measured CSF sTREM2 in AD patients and found that sTREM2 is slightly increased. Suárez-Calvet et al. [112] measured the level of CSF TREM2 in subjects with preclinical AD, mild cognitive impairment due to AD (MCI-AD), and AD case-controls and found that the levels of CSF sTREM2 dynamically change during the AD process and peak at MCI-AD patients. Similarly, Matarin et al. [113] found that the gene expression of TREM2, accompanied by microglia activation, is significantly increased at 18 months in AD mice with tau pathology. Another interesting finding is that the increased CSF sTREM2 levels in these tauopathy mice were associated with higher CSF phospho-tau, which significantly contributed to synaptic degeneration [114]. Based on these findings, it can be anticipated that sTREM2 probably reflects a corresponding change in microglial function in response to synaptic degeneration in the early stage of AD.

It will be very helpful to learn whether microglia principally participate in the plaque formation or tau-related pathology of AD; however, it is generally confirmed that microglia-associated neuroinflammation may directly contribute to the initiation of synaptic and AD-like cognitive impairment. Therefore, it is time to focus on the investigation of the early stage AD processes and mechanisms of the microglia-synapse pathway that contribute to the pathogenesis of AD.

## 6. Microglia-Synapse Pathways for AD Therapy

The mechanisms underlying the neuropathology of AD remain unclear. Current therapeutic strategies for AD are mainly limited to ineffective symptomatic treatments, but some of the available disease-modifying drugs also perform poorly. Among the disease-modifying drugs, most of the trials involve passive immunosuppression. However, so far, nearly all of the clinical trials have failed, which leads to the initiation of clinical trials targeting patients at the presymptomatic or early stages of AD. Increasingly, microglia-synapse pathways have been confirmed as a promising strategy for early stage AD treatment.

It has been demonstrated that depletion of NLRP3 could significantly suppress amyloidosis and neuropathology, as well as improve cognitive function in AD mice [35], indicating that the NLRP3 inflammasome is a possible molecular target for neuroprotection and therapeutic intervention in AD. Agents for handling the activation of the NLRP3 inflammasome in microglia may offer considerable promise to block neuroinflammation and slow the progression of AD. The type 2 diabetes drug glyburide also prevents activation of the NLRP3 inflammasome [115]. More importantly, the antimalarial drug artemisinin was recently shown to exert protective effects in AD pathology via inhibiting the activation of NF-*κ*B and the NLRP3 inflammasome in AD mice [116], further verifying that targeting the NLRP3 inflammasome may offer a crucial intervention for AD progression.

Mdivi-1, a noncompetitive inhibitor of Drp1 GTPase activity that attenuates Drp1-mediated mitochondrial-fission and responds to the stimulation of proapoptotic signaling [117], has been developed as a therapeutic agent for AD. Mdivi-1 also partially rescues the mitochondrial damage due to inactivation of PTEN-induced putative kinase 1 (PINK1)/Parkin pathway, which modulates mitophagy [118]. AD-related mitochondrial stress effectively triggers Parkin-dependent mitophagy, which establishes the need for further investigation of the regulation of mitophagy to potentially ameliorate mitochondrial dysfunction within synapses in AD. Future research should focus on targeting therapeutics toward preserving mitochondrial function as a treatment for AD.

A recent study showed that synaptic dysfunction in cognitively normal AD mice was due to proinflammatory factors [119]. It has also been reported that maze performance and dendritic spine density improved following the removal of most microglia [120] or one subset of dysfunctional microglia [121] in AD mice by pharmacological inhibition of colony-stimulating factor 1 receptor (CSFR1) for 1 month. Since elimination of microglia was shown to impair learning [29], inducing normal microglia may be a more effective therapeutic strategy. Microglia can self-renew without peripheral aid [122], and the repopulated microglial niche may be functional and could potentially provide further benefit in AD brains, especially if the microglia have never encountered A*β*, tau, or other AD-related factors. Moreover, improving microglial phagocytosis via PPAR-receptor agonism-dependent induction of CD36 was found to stimulate A*β* clearance and enhance spatial memory [123]. However, not all phagocytosis is inherently good or bad, and it is important to simultaneously prevent C1qa-dependent synapse loss while promoting A*β* phagocytosis [25].

There is still no available therapy that can successfully reverse AD, but the current evidence suggests that the best treatment for AD may consist of a therapeutic strategy combined with anti-inflammatory factors. Therapy for AD faces many challenges with respect to timing and efficacy, and targeting the proinflammatory mediators may not be effective. However, early stage intervention targeting dysregulated proinflammatory mediators might be therapeutically beneficial. A small molecule named MW-151 has been tested during two distinct therapeutic temporal windows at the early stage of the disease in APP/PS1 transgenic mice. MW-151 treatment attenuates microglial activation and the production of proinflammatory cytokines in the cortex, which protects against the synaptic dysfunction implicated in learning and memory deficits [124]. Targeting the balance of M1/M2 phenotypes in microglia depends on the temporal window, since the stages of AD are associated with changes in the microglial activation states. Anti-inflammatory drugs will need the ability to access the CNS, and the chemicals fasudil [125] and minocycline [126] both have the ability to effectively cross the blood-brain barrier, which was demonstrated to enhance the anti-inflammatory abilities of M2 microglia. Recently, replacement of diseased neurons with new neurons derived from embryonic stem cells or pluripotent stem cells was shown to have a beneficial clinical outcome.

Promisingly, nervous system growth factors can prevent neuronal loss in AD animal models. Previous studies revealed that nerve growth factor (NGF) can stimulate cholinergic neurons associated with cognition and prevent their impairment in early stage AD. However, systemic administration of NGF can cause adverse side effects, requiring a targeted delivery strategy to control the localization and spread in the brain. In a phase I trial, NGF was verified to provide beneficial effects over a two-year observation period. Another phase I clinical trial enrolled 10 patients who received AAV2-NGF. NGF induced axonal sprouting toward the NGF, lowered the rate of cognitive decline, and increased cortical glucose uptake. Glial cell-derived neurotrophic factor (GDNF) is a neurotrophic factor with therapeutic potential for AD. It was reported that 3xTg-AD mice showed preserved learning and memory with 6 months of GDNF overexpression. GDNF therapy did not significantly reduce amyloid or tau pathology, but it upregulated the expression of BDNF and induced neuroprotection. In mice with Atg7-deficient microglia, the loss of autophagy in early developmental microglia impaired synaptic pruning and increased dendritic spine density, suggesting that microglial autophagy has an important role in regulating synaptic homeostasis and neuropsychological behaviors. IL-4 has also been characterized as a potential modulator of neuronal activities in the brain. IL-4 receptors are expressed in the hippocampus, and downregulation of IL-4 causes aging-related deficits in hippocampal LTP. Anti-inflammatory cytokines such as IL-10 may also have significant therapeutic potential for the treatment of AD. Neuronal expression of the mouse IL-10 gene ameliorates cognitive dysfunction in APP/PS1 transgenic mice [127].

## 7. Perspectives

**Figure 1:**
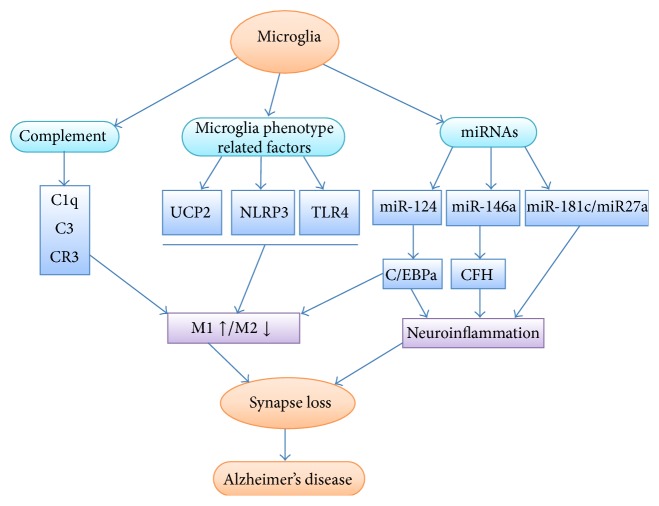
Schematic representation of possible mechanisms of the microglia-synapse pathways in AD. Microglial phenotype could switch from an immunosuppressive M2 phenotype into a proinflammatory M1 phenotype through complement pathways and several crucial factors, such as UCP2, NLRP3, and TLR4. The phenotype transition and neuroinflammation by related miRNAs induce synapse loss in AD pathogenesis.

Recently, in the AD etiology literature, considerable attention has been paid to the emerging specialized functions of microglia in regulating synaptic development and degeneration, as synapse loss is the most directly relevant pathology to the development of cognitive deficits in AD. As shown in Figure 1, many studies have repeatedly identified the microglia-synapse pathway as the crucial factor in AD pathogenesis. In future studies, how microglia respond to their environment and switch their phenotypes following the early stages of an insult should be determined in both the infant and aging brain, because current evidence shows that microglia with normal cellular homeostasis become aberrant and dysregulated during the aging process of the CNS, resulting in an increased susceptibility to subsequent immune challenges. In addition, genetic approaches should be applied to explore key targets contributing to the connections or activation of the microglia-synapse pathways to achieve early prevention and a potential cure for AD.
